# Diagnostic Accuracy of the Thyroid Imaging Reporting and Data System (TI-RADS) and the Bethesda System: Correlation With Final Histopathology

**DOI:** 10.7759/cureus.108317

**Published:** 2026-05-05

**Authors:** Haneen Alboosta, Noora Fuad Ali, Reem Althawadi, Reem AlBuainain, Ahmed J Isa, Ahmed A Alkazaz, Zahraa Radhi, Israa Abualainain, Ahmed AlSharakhat, Hamza Muneer

**Affiliations:** 1 General Surgery, Salmaniya Medical Complex, Manama, BHR; 2 General Surgery, Salmanyia Medical Complex, Manama, BHR; 3 Plastic Surgery, Salmaniya Medical Complex, Manama, BHR; 4 Pathology, Salmaniya Medical Complex, Manama, BHR

**Keywords:** benign and malignant thyroid nodules, bethesda reporting system, bethesda system for reporting thyroid cytopathology, fine needle aspiration cytology (fnac), thyroid cancer, thyroid imaging reporting and data system, ti-rads

## Abstract

Background

Thyroid cancer is the most common malignancy of the head and neck, resulting in a high volume of referrals for evaluation of thyroid nodules. Ultrasound risk stratification using the Thyroid Imaging Reporting and Data System (TI-RADS) and cytological assessment via the Bethesda System for Reporting Thyroid Cytopathology are central to preoperative decision-making. However, their diagnostic performance varies when validated against final histopathology.

Objectives

The primary objective of this study was to assess the concordance between TI-RADS, Bethesda cytology, and final histopathology in surgically managed patients. The secondary objective was to evaluate preoperative malignancy detection rates and identify missed malignancies or discrepant cases.

Methods

This retrospective observational study included patients who underwent total or hemithyroidectomy at Salmaniya Medical Complex, Manama, Bahrain, between 2017 and 2024. Preoperative TI-RADS and Bethesda classifications, demographic data, and nodule characteristics were analyzed. Diagnostic performance was assessed for both modalities and further stratified by histological subtype, indeterminate categories (TI-RADS 3-4, Bethesda III-IV), tumor size, age, and sex.

Results

Of 594 patients who underwent thyroid surgery, 159 cases with histologically confirmed malignancy and complete preoperative data were included in the final analysis (mean age: 44.7 years; females: 127 (79.9%)). Ultrasound correctly identified 137 (86.2%) of malignant nodules, while fine needle aspiration cytology (FNAC) detected 134 (84.3%). Both modalities demonstrated reduced detection rates for follicular carcinoma, with ultrasound identifying seven of 10 cases (70.0%) and cytology identifying six of 10 cases (60.0%). Malignancy was observed within indeterminate and low-risk categories, including TI-RADS 3 nodules and Bethesda III cytology. Among Bethesda III nodules (n= 52), 15 cases (28.8%) were malignant on final histopathology. Additionally, a substantial proportion of benign nodules classified as high-risk on ultrasound (TI-RADS ≥4) exhibited benign or indeterminate cytology. Larger tumors (>4 cm) were more reliably detected, whereas age, sex, and tumor location did not significantly affect diagnostic performance.

Conclusion

Ultrasound TI-RADS and Bethesda cytology demonstrate high but imperfect sensitivity for the preoperative detection of thyroid malignancy, with ultrasound showing slightly superior performance. Diagnostic limitations were most evident in follicular-pattern tumors and indeterminate nodules. These findings emphasize the importance of integrated ultrasound and cytological assessment to improve risk stratification and guide surgical decision-making in patients with thyroid nodules.

## Introduction

Thyroid cancer is the most common endocrine malignancy worldwide [1]. A thyroid nodule is defined as a discrete lesion within the thyroid gland that is radiologically distinct from the surrounding parenchyma [2]. Although the majority of thyroid nodules are benign and often discovered incidentally, the primary objective of clinical evaluation is to exclude malignancy [3]. Clinically palpable nodules are identified in approximately 5% of the general population, while high-resolution ultrasonography detects thyroid nodules in up to 68% of individuals, frequently measuring only a few millimeters to 1 cm in size [3, 4].

The risk of malignancy in thyroid nodules is influenced by a combination of genetic, environmental, and clinical factors [4, 5]. Childhood exposure to ionizing radiation and familial thyroid cancer syndromes are associated with the highest individual risks, with reported malignancy rates reaching 35%-40% [1, 3-5]. Additional predictors include advanced age, female sex, compressive symptoms, and the presence of suspicious cervical lymphadenopathy [1, 3-5]. Despite the high prevalence of thyroid nodules, population-based studies estimate that only 5%-13% harbor malignancy [1, 3-7]. This disparity underscores the importance of a structured, evidence-based approach to diagnostic evaluation to avoid unnecessary procedures while ensuring timely cancer detection [3, 5-10].

Standard evaluation of thyroid nodules typically includes thyroid function testing and high-resolution neck ultrasound, followed, when indicated, by ultrasound-guided fine needle aspiration cytology (FNAC). To standardize ultrasound-based risk stratification, the Thyroid Imaging Reporting and Data System (TI-RADS) was developed to categorize nodules according to specific sonographic features, assigning risk levels from TR1 to TR5 that correspond to increasing malignancy probability [11]. These categories inform clinical decisions regarding surveillance, biopsy, or no further evaluation. Cytological assessment of biopsied nodules is commonly reported using the Bethesda System for Reporting Thyroid Cytopathology, which classifies findings into six diagnostic categories ranging from non-diagnostic to malignant, each associated with an estimated malignancy risk [5].

Recent clinical guidelines emphasize the integration of ultrasound risk stratification and cytological findings in the management of thyroid nodules, with additional consideration of molecular testing in selected cases [12]. While numerous studies have independently examined the diagnostic performance of TI-RADS, Bethesda cytology, and postoperative histopathology, direct comparison of all three modalities within the same surgically treated patient cohort remains limited. Furthermore, the performance of these systems in indeterminate categories and in identifying malignancies that are missed preoperatively has not been fully characterized.

This study aims to evaluate the diagnostic concordance between ultrasound TI-RADS and Bethesda cytology using final histopathological diagnosis as the reference standard and to identify diagnostic inconsistencies and clinicopathological features associated with missed malignancies in a surgically treated population.

## Materials and methods

Design and setting

This retrospective observational study was conducted at Salmaniya Medical Complex, Manama, the largest tertiary care referral center in Bahrain. Medical records of patients managed by the General Surgery Department between January 2017 and December 2024 were reviewed to evaluate the diagnostic concordance between ultrasound-based TI-RADS, preoperative FNAC reported using the Bethesda System, and final postoperative histopathology.

Study population

The study cohort consisted of patients evaluated for thyroid nodules at Salmaniya Medical Complex who underwent preoperative ultrasound, FNAC, and subsequent surgical management. Both benign and malignant nodules confirmed on final histopathology were included in the overall cohort analysis. Subgroup analyses focusing on diagnostic performance and missed malignancies were performed in patients with histologically confirmed thyroid cancer.

Patients were excluded if any component of the diagnostic pathway (ultrasound, FNAC, or surgery) was incomplete or if any part of the preoperative evaluation was performed outside Salmaniya Medical Complex, resulting in unverifiable records. After applying these criteria, 159 patients with histologically confirmed malignancy were included in the final analytical subgroup.

Data collection

Data were extracted from the institutional electronic medical record system (I-SEHA). Collected variables included patient demographics (age and sex), nodule characteristics (size, number, and anatomical location), ultrasound TI-RADS classification, FNAC Bethesda category, and final histopathological diagnosis.

Statistical analysis

Diagnostic performance was assessed by categorizing TI-RADS 1-2 and Bethesda categories I-II as benign (missed malignancy), and TI-RADS 3-5 and Bethesda categories III-IV as malignant (detected malignancy). Bethesda category 1 was grouped in the “missed malignancies” for analytical purposes, as non-diagnostic cytology does not provide pre-operative detection of malignancies. Bethesda categories III-VI and TI-RADS 3 were labelled as “Detected”, again for study purposes, as they allow the identification of nodules that require further workup. Sensitivity and proportions of detected and missed malignancies were calculated for each diagnostic modality. Associations between categorical variables were analyzed using the chi-square (χ²) test, where appropriate. A p-value of <0.05 was considered statistically significant. Statistical analyses were performed using standard descriptive and inferential methods.

Ethical approval

This study was conducted in accordance with the principles of the Declaration of Helsinki and was approved by the Research and Ethics Committee at Salmaniya Medical Complex (approval number: 119-301225). All data were anonymized prior to analysis, and the requirement for informed consent was waived by the institutional review board due to the retrospective nature of the study.

## Results

A total of 594 patients underwent initial thyroid ultrasonography, FNAC, and thyroid surgery at Salmaniya Medical Complex from 2017 to 2024. Final histopathological examination confirmed malignancy in 262 cases, while 332 cases were benign. Of the malignant cases, 159 patients were included in the final analysis, and 103 cases were excluded due to incomplete clinical or cytological data or because the preoperative diagnostic workup was performed outside our institute, resulting in non-verifiable records.

**Figure 1:**
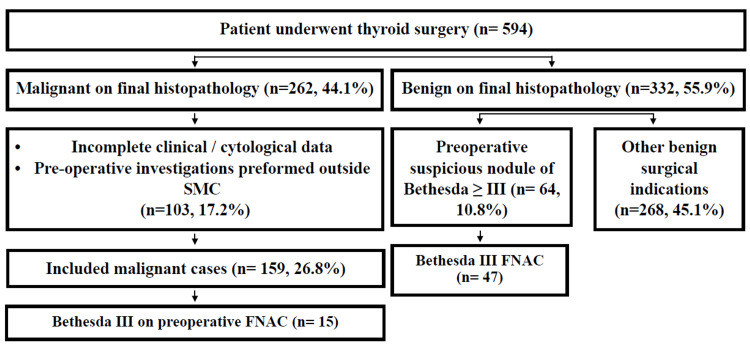
Flow diagram illustrating patient selection and inclusion in the final study cohort SMC: Salmaniya Medical Complex; FNAC: fine needle aspiration cytology Figure created by the authors using Microsoft Word (Microsoft Corp., Redmond, WA, USA).

Within the benign cohort (332 cases), 64 patients underwent surgery for cytologically suspicious nodules (Bethesda III or higher); of these, 47 had preoperative Bethesda III cytology, all of which were subsequently confirmed benign on final histopathology. The remaining benign cases underwent thyroid surgery for other non-malignant indications (Figure 1).

Among all nodules confirmed benign on final histopathology, a substantial proportion of lesions classified as high-risk on ultrasound TI-RADS ≥4 demonstrated low or indeterminate cytology (Bethesda III or lower). Specifically, within TI-RADS 4 nodules (n = 53), 43 (81.1%) cases were categorized as Bethesda II or III, while only 10 (18.9%) cases were Bethesda IV or V. Similarly, among TI-RADS 5 nodules (n = 24), 17 (70.8%) cases were Bethesda III or lower. Overall, 60 of 77 (77.9%) nodules classified as TI-RADS ≥4 corresponded to Bethesda III or lower cytology, despite all lesions being benign on final histopathology (Table 1).

**Table 1:** Distribution of Bethesda cytology categories across Ti-RADS classifications in benign thyroid nodules TI-RADS: Thyroid Imaging Reporting and Data System

Bethesda category (n)	TI-RADS 2	TI-RADs 3	TI-RADS 4	TI-RADS 5
Bethesda II (98)	32	41	19	6
Bethesda III (47)	3	10	23	11
Bethesda IV (12)	0	4	10	3
Bethesda V (5)	0	0	1	4

A total of 52 nodules were classified as Bethesda III on preoperative cytology. On final histopathology, 15 (28.8%) nodules were malignant, and 47 (71.2%) nodules were benign, after exclusion of predefined excluded cases. Among the 47 benign Bethesda III nodules, ultrasound classified 13 (27.7%) nodules as TI-RADS ≤3 and 34 (72.3%) nodules as TI-RADS ≥4, including 23 TI-RADS 4 and 11 TI-RADS 5 lesions.

The results of the included 159 patients are summarized in Table 2. The cohort had a mean age of 44.72 years (range: 19-79 years), and females accounted for 127 (79.9%) of cases. Thyroid lesions were observed in both single and multiple sites across the gland. Histologically, the classical papillary carcinoma subtype was most prevalent, accounting for 117 (73.6%) cases. Imaging findings revealed that TI-RADS 5 nodules were most frequently encountered (70 (44%)), while Bethesda category VI dominated the FNAC results (64 (40.3%)).

**Table 2:** Demographic, clinical, and preoperative diagnostic characteristics of patients with histologically confirmed thyroid malignancy (n = 159) US: ultrasound; TI-RADS: Thyroid Imaging Reporting and Data System; FNAC: fine needle aspiration cytology

Variable	Category/Value	N (%)/Statistic
Age (years)	Range	19-79
	Mean	44.72
	Median (IQR)	45 (36-53)
Gender	Male	32 (20.1%)
	Female	127 (79.9%)
Lesion site	Right	51 (32.1%)
	Left	42 (26.4%)
	Isthmus	4 (2.5%)
	Combined sites	62 (39%)
Type of carcinoma	Papillary, classical	117 (73.6%)
	Papillary, follicular variant	26 (16.4%)
	Medullary	5 (3.1%)
	Follicular	10 (6.3%)
	Anaplastic	1 (0.6%)
US TI-RADS	5	70 (44%)
	4	47 (29.6%)
	3	20 (12.6%)
	2	21 (13.2%)
	1	1 (0.6%)
FNAC Bethesda	6	64 (40.3%)
	5	47 (29.6%)
	4	8 (5%)
	3	15 (9.4%)
	2	23 (14.5%)
	1	2 (1.3%)

When categorized for diagnostic performance (Table 3), TI-RADS 1-2 and Bethesda I-II were considered benign (missed), while TI-RADS 3-5 and Bethesda III-VI were classified as malignant (detected). Using these criteria, ultrasound TI-RADS correctly identified 137 (86.2%) cases preoperatively, while 22 (13.8%) cases were missed. FNAC identified 134 (84.3%) cases, missing 25 (15.7%) cases. Thus, ultrasound slightly outperformed FNAC for preoperative detection of malignancy, although both methods missed a small proportion of confirmed cases.

**Table 3:** Preoperative diagnostic performance of ultrasound TI-RADS and FNAC in detecting thyroid malignancy (n = 159)* *All cases were histologically confirmed malignant cases. **Categories 3–5 for TI-RADS and III–VI for Bethesda were considered malignant (detected). ***TI-RADS categories I–II and Bethesda categories I-II were considered benign (missed). TI-RADS: Thyroid Imaging Reporting and Data System; FNAC: fine needle aspiration cytology

Modality	Malignant detected**	Malignant missed***	Detection rate (%)
Ultrasound (TI-RADS 3–5)	137	22	86.2
FNAC (Bethesda III-VI)	134	25	84.3

**Figure 2:**
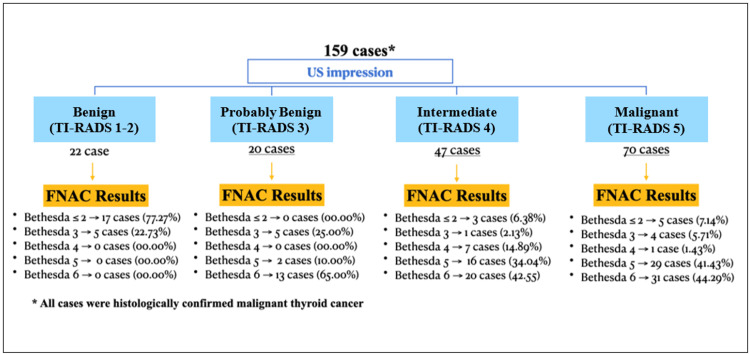
Correlation between US TI-RADS categories and FNAC Bethesda System classifications in histologically confirmed thyroid malignancies *All cases were histologically confirmed malignant thyroid cancer US: ultrasound; TI-RADS: Thyroid Imaging Reporting and Data System; FNAC: fine needle aspiration cytology Figure created by the authors using Microsoft Word (Microsoft Corp., Redmond, WA, USA).

Analysis of the correlation between TI-RADS and FNAC Bethesda categories demonstrated a positive relationship (Table 4, Figure 2). Increasing ultrasound risk (higher TI-RADS grade) was consistently associated with higher-risk cytological findings. For instance, most TI-RADS 5 nodules (n = 70) corresponded to the highest-risk Bethesda classes (V and VI), while lower TI-RADS grades were more distributed across intermediate Bethesda categories. This supports the clinical utility of ultrasound-based TI-RADS grading for predicting cytological malignancy suspicion.

**Table 4:** Association and agreement between TI-RADS and Bethesda System classification US: ultrasound; TI-RADS: Thyroid Imaging Reporting and Data System

Analysis	Statistics	Value
Association	Chi-square (χ², df)	194.3 (20)
	P-value	< 0.001
Overall agreement	Cohen’s kappa (κ)	0.229 (fair agreement)
Correlation	Spearman’s rho (ρ)	0.340
	P-value	< 0.001
	Pearson’s r	0.521
	P-value	p < 0.001
Association between US TI-RADS and Bethesda categories was assessed using the chi-square test. Agreement beyond chance was evaluated using Cohen’s kappa. Spearman’s correlation coefficient was used to assess ordinal correlation. A p-value < 0.05 was considered statistically significant		

When stratified by histological subtype, both ultrasound and FNAC demonstrated high preoperative sensitivity across most tumor types, but minor variations were observed (Table 5). Classical type papillary carcinoma achieved the highest detection rates, with ultrasound identifying 103 of 117 (88.0%) cases and FNAC identifying 100 of 117 (85.5%) cases, followed by the follicular variant (22 of 26 cases (84.6%) by ultrasound and 21 of 26 cases (80.8%) by FNAC). Follicular carcinoma was more frequently under-detected, with detection rates of seven of 10 cases (70.0%) by ultrasound and six of 10 cases (60.0%) by FNAC. Medullary carcinoma showed consistent detection, with four of five cases (80.0%) identified by both modalities, while all cases of anaplastic carcinoma (one of one (100%)) were correctly identified as malignant by both methods. Overall, both diagnostic methods provided preoperative sensitivity exceeding 80% for malignancy detection, with only minor discrepancies across specific histological subtypes (Table 6).

**Table 5:** Detection rates of thyroid malignancy by ultrasound TI-RADS and FNAC according to histological subtypes US: ultrasound; TI-RADS: Thyroid Imaging Reporting and Data System; FNAC: fine needle aspiration cytology

Histological subtype	Detected by US TI-RADS ≥3	Detected by FNAC Bethesda ≥III
Papillary carcinoma, classical (n=117)	103 (88.0%)	100 (85.5%)
Papillary carcinoma, follicular variant (n=26)	22 (84.6%)	21 (80.8%)
Follicular carcinoma (n=10)	7 (70.0%)	6 (60.0%)
Medullary carcinoma (n=5)	4 (80.0%)	4 (80.0%)
Anaplastic carcinoma (n=1)	1 (100%)	1 (100%)

**Table 6:** Subgroup analysis of agreement between US TI-RADS and Bethesda System categories -: not statistically significant; US: ultrasound; TI-RADS: Thyroid Imaging Reporting and Data System

Variable	Subgroup	p-value	Cohen’s Kappa (κ)
Age	< 55 years	< 0.001	0.246
	≥ 55 years	0.004	0.166
Gender	Male	< 0.05	0.227
	Female	< 0.001	0.229
Lesion site	Right lobe	< 0.001	0.292
	Left lobe	< 0.001	0.263
	Isthmus / Combined	-	-
Histological type	Papillary classical	< 0.001	0.236
	Papillary follicular variant	0.028	0.157
	Follicular carcinoma	0.014	0.394
	Medullary carcinoma	-	-
	Anaplastic carcinoma	NA	-

Tumor size influenced the accuracy of detection by ultrasound TI-RADS and Bethesda cytology (Table 7). Microcarcinomas (≤1 cm, n = 45) were more likely to be classified as TI-RADS 2-3, with wide variability in Bethesda categories, indicating less reliable detection (p = 0.094). Larger tumors (>4 cm, n = 21) were detected more consistently by both methods, with statistically significant associations (p < 0.001). Agreement analysis demonstrated fair and statistically significant agreement in microcarcinomas and intermediate-size tumors (κ = 0.300 and κ = 0.240, respectively), whereas agreement remained poor in large tumors (κ = 0.118). Stratified analysis of other factors, including age, gender, and tumor site, showed no significant impact on detection accuracy. Overall, detection reliability improved with increasing tumor size, although some discrepancies in classification remained.

**Table 7:** Detection accuracy and agreement between US TI-RADS and Bethesda System by tumor size US: ultrasound; TI-RADS: Thyroid Imaging Reporting and Data System

Tumor size	n	Cohen’s kappa (κ )	Interpretation	p-value	Interpretation
≤ 1 cm (sizeTNM 0)	45	0.300	Fair agreement	0.094	Weak evidence
> 1 cm <4	92	0.240	Fair agerement	0.004	Moderate evidecne
> 4	21	0.118	Poor agreement	< 0.001	Very strong evidence

## Discussion

In this single-center retrospective study of 159 patients with histologically confirmed thyroid malignancy, both American College of Radiology (ACR) TI-RADS and Bethesda cytology demonstrated good preoperative diagnostic performance, correctly identifying malignancy in 86.2% and 84.3% of cases, respectively. A positive correlation between increasing TI-RADS categories and higher-risk Bethesda classifications was observed, supporting a reasonable level of concordance between ultrasound-based risk stratification and cytological assessment in routine clinical practice.

This finding is consistent with existing literature reporting moderate to strong agreement between TI-RADS and cytology, particularly for papillary thyroid carcinoma (PTC), which constituted the predominant histological subtype in our cohort. TI-RADS performs particularly well in PTC due to the incorporation of characteristic sonographic features such as hypoechogenicity, microcalcifications, irregular margins, and a taller-than-wide shape into its scoring framework. In contrast, follicular thyroid carcinoma remains a diagnostic challenge in the preoperative setting. The defining features of follicular carcinoma, capsular and vascular invasion, as defined by the World Health Organization, cannot be reliably assessed by ultrasound or cytology and require histopathological examination, explaining the lower detection rates observed for this subtype in our study and others [13, 14].

Tumor size significantly influenced diagnostic accuracy in our cohort. Microcarcinomas were more frequently under-detected or classified into lower TI-RADS and Bethesda categories, whereas larger tumors were identified more consistently by both modalities. This finding reflects a well-recognized limitation of ultrasound and cytology in very small lesions and highlights the importance of integrating imaging and cytological findings with clinical context, rather than relying on a single diagnostic tool, particularly in patients undergoing surgical evaluation.

Although TI-RADS demonstrated a tendency toward higher-risk categorization compared with Bethesda cytology in malignant cases, a similar pattern was observed among nodules with benign final histopathology. This suggests that ultrasound risk overestimation is not limited to malignant lesions and reflects the design of TI-RADS as a sensitive screening and triage tool. Ultrasound-based systems prioritize sensitivity to avoid missed malignancies and to guide appropriate biopsy referral, which may result in higher risk categorization for nodules that ultimately prove benign due to overlapping sonographic features and limited specificity [15, 16]. Current guideline-based reviews continue to support the use of TI-RADS for biopsy decision-making while acknowledging its inherent limitations in specificity [17].

Our findings are consistent with previous studies showing that both thyroid ultrasound and FNAC are influenced by operator experience and local institutional practice [18-20]. An institutional study by Nebu et al. reported superior performance of FNAC compared with ultrasound [18]. In their cohort, it remained a useful predictor of malignancy in indeterminate thyroid nodules, correctly identifying a substantial proportion of malignant cases preoperatively. Based on these findings, the authors suggested that ultrasound-TIRADS may be considered as a practical risk-stratification tool in low-resource settings, where access to repeat FNAC, molecular testing, or advanced diagnostics may be limited [18].

Supporting this approach, a recent comparative study evaluating ultrasound TI-RADS and FNAC demonstrated a significant correlation between higher TI-RADS categories and more suspicious cytological and histopathological outcomes, indicating that standardized ultrasound classification can enhance diagnostic concordance when applied within structured institutional protocols [19]. Current clinical guidelines emphasize that standardized ultrasound reporting, consistent biopsy thresholds, and multidisciplinary decision-making can help reduce this variability and improve concordance between imaging and cytology [21]. In addition, meta-analyses similarly support the use of TI-RADS as a reliable risk-stratification tool, especially when ultrasound findings are interpreted alongside cytological results within structured diagnostic pathways [22].

A particularly important finding in our study relates to indeterminate cytology. In our cohort, Bethesda III nodules demonstrated a malignancy rate of 15 of 52 nodules (28.8%) on final histopathology. This exceeds the original malignancy risk estimate of 5%-15% proposed in the Bethesda System for Reporting Thyroid Cytopathology but is consistent with more recent evidence suggesting higher malignancy rates in this category. The 2023 revision of the Bethesda System acknowledges this trend, reporting an updated estimated malignancy risk of approximately 22% for Bethesda III nodules [23].

Several contemporary surgical series from high-volume centers have reported malignancy rates for Bethesda III nodules ranging from 26.6% to 37.8%, particularly when including both atypia of undetermined significance and follicular lesion of undetermined significance subcategories [24]. In surgically selected cohorts, reported malignancy rates have reached 31.2%-38.7%, reflecting referral and selection bias toward higher-risk nodules [23]. Other large multi-institutional analyses have demonstrated malignancy rates approaching 32% in Bethesda III nodules, with some tertiary care series reporting even higher rates [23]. The variability in reported malignancy rates likely reflects differences in cohort composition, inclusion criteria, and institutional practice patterns; however, collectively, these findings support that the malignancy risk associated with Bethesda III nodules frequently exceeds historical estimates. Our observed malignancy rate of approximately 30%, therefore, aligns closely with contemporary surgical literature and underscores the clinical significance of this indeterminate category.

In the Bahraini context, a prior single-center study evaluated the correlation between ultrasound TI-RADS and Bethesda cytology without access to final histopathological outcomes [25]. That study demonstrated a significant association between ultrasound risk categories and cytological findings, supporting concordance between the two diagnostic modalities. Our study builds upon this local experience by incorporating final surgical histopathology as the reference standard, allowing assessment of true diagnostic performance and identification of missed malignancies and discordant cases. Together, these studies provide complementary insights into thyroid nodule evaluation across tertiary care settings in Bahrain and highlight the added value of histopathological validation in surgical cohorts.

Overall, our findings support the complementary use of ultrasound TI-RADS and FNAC Bethesda systems in the evaluation of thyroid nodules. While both modalities demonstrate good diagnostic performance, limitations persist, particularly in follicular-pattern tumors, microcarcinomas, and indeterminate cytology. These results underscore the importance of integrated interpretation of imaging and cytological findings, adherence to standardized diagnostic pathways, and continued multicenter research to refine risk stratification and improve diagnostic consistency in clinical practice.

Limitations

This study has several important limitations. Its retrospective, single-center design may limit generalizability to other institutions and healthcare settings. Although both benign and malignant nodules were included, the study cohort represents a surgically treated population, which introduces referral and selection bias toward higher-risk nodules and may not reflect the full spectrum of thyroid nodules encountered in screening or outpatient settings. As a result, malignancy rates, particularly within indeterminate categories, may be higher than those observed in non-surgical cohorts. In addition, because the indeterminate categories “TI-RADS 3 and Bethesda III” were included in the “detected” group, this could have overestimated diagnostic sensitivity.

Operator dependence represents another potential source of variability. Ultrasound interpretation and FNAC sampling and reporting are subject to inter-observer differences, which may influence TI-RADS categorization and Bethesda classification despite the use of standardized reporting systems. Variability in image acquisition, cytological adequacy, and interpretation cannot be fully excluded. 

Furthermore, a considerable proportion of malignant cases have been excluded due to either incomplete investigations or being performed externally without verifiable standardized records, which could have introduced selection bias.

Our findings are also most applicable to surgically selected or high-risk tertiary care centers and, hence, should not be generalized to an unselected outpatient group.

Finally, while the overall sample size was adequate for descriptive analyses and primary comparisons, the number of cases within certain histological subtypes and tumor size subgroups was relatively small, limiting statistical power for detailed subgroup analyses. Future prospective multicenter studies incorporating standardized imaging protocols, broader nodule populations, and molecular testing where available may help address these limitations and further refine risk stratification strategies.

## Conclusions

In this surgically treated cohort, ultrasound-based ACR TI-RADS demonstrated good concordance with Bethesda cytology and final histopathology, with diagnostic performance comparable to FNAC for preoperative detection of thyroid malignancy. Increasing TI-RADS categories correlated with higher-risk cytological classifications, supporting the complementary role of ultrasound and cytology in thyroid nodule evaluation. However, these conclusions primarily apply to preoperative diagnostic performance and concordance within high-risk patients.

Clinically significant malignancies were also observed within indeterminate and lower-risk categories, particularly TI-RADS 3 nodules and Bethesda III cytology, and both diagnostic modalities showed reduced sensitivity for follicular-pattern tumors and very small lesions. These findings highlight important diagnostic limitations and reinforce the need for integrated interpretation of imaging, cytology, and clinical context rather than reliance on a single modality.

Overall, our results support the continued use of TI-RADS as a practical, noninvasive tool to guide FNAC referral and surgical decision-making, while emphasizing its role as part of a multimodal diagnostic strategy. Future prospective multicenter studies, including both surgical and non-surgical populations, with standardized imaging protocols and incorporation of molecular testing where available, are needed to further refine risk stratification and optimize diagnostic pathways in routine clinical practice.
